# Dysregulation of cellular iron metabolism in Friedreich ataxia: from primary iron-sulfur cluster deficit to mitochondrial iron accumulation

**DOI:** 10.3389/fphar.2014.00130

**Published:** 2014-06-03

**Authors:** Alain Martelli, Hélène Puccio

**Affiliations:** ^1^Department of Translational Medecine and Neurogenetics, Institut de Génétique et de Biologie Moléculaire et CellulaireIllkirch, France; ^2^INSERM, U596Illkirch, France; ^3^CNRS, UMR7104Illkirch, France; ^4^Université de StrasbourgStrasbourg, France; ^5^Chaire de Génétique Humaine, Collège de FranceIllkirch, France

**Keywords:** Friedreich ataxia, frataxin, iron metabolism, iron-sulfur cluster, mitochondria, iron metabolism disorders

## Abstract

Friedreich ataxia (FRDA) is the most common recessive ataxia in the Caucasian population and is characterized by a mixed spinocerebellar and sensory ataxia frequently associating cardiomyopathy. The disease results from decreased expression of the FXN gene coding for the mitochondrial protein frataxin. Early histological and biochemical study of the pathophysiology in patient's samples revealed that dysregulation of iron metabolism is a key feature of the disease, mainly characterized by mitochondrial iron accumulation and by decreased activity of iron-sulfur cluster enzymes. In the recent past years, considerable progress in understanding the function of frataxin has been provided through cellular and biochemical approaches, pointing to the primary role of frataxin in iron-sulfur cluster biogenesis. However, why and how the impact of frataxin deficiency on this essential biosynthetic pathway leads to mitochondrial iron accumulation is still poorly understood. Herein, we review data on both the primary function of frataxin and the nature of the iron metabolism dysregulation in FRDA. To date, the pathophysiological implication of the mitochondrial iron overload in FRDA remains to be clarified.

## Introduction

Ataxias are a heterogeneous group of disorders characterized by loss of coordination due to the degeneration of the neuronal networks closely linked to cerebellar function. Friedreich's ataxia (FRDA) is the most prevalent form of hereditary ataxia in Caucasians, accounting for 75% of ataxia with onset prior to 25 years of age (Cossee et al., 1997). FRDA is characterized by progressive spinocerebellar and sensory ataxia (Harding, 1981). The symptoms associated with the disease include the absence of deep tendon reflexes, dysarthria, pyramidal signs, muscular weakness, and positive extensor plantar response (Harding, 1981; Pandolfo, 2009). The neurological symptoms result from progressive degeneration of large sensory neurons in the dorsal root ganglia (DRG) and their axonal projection in the posterior columns, as well as from degeneration of the spinocerebellar and corticospinal tracts of the spinal cord (Koeppen and Mazurkiewicz, 2013). The dentate nucleus of the cerebellum is also affected and accounts for the cerebellar phenotype (Koeppen, 2011). FRDA is also characterized by primary non-neurological manifestations, in particular hypertrophic cardiomyopathy and increased incidence of diabetes (Harding and Hewer, 1983). The cardiomyopathy associated with FRDA is due to the natural transition from hypertrophy to dilation. The latter promotes cardiomyocytes death and replacement of contractile cells by fibrotic tissue leading to severe systolic and diastolic dysfunction (Tsou et al., 2011; Payne and Wagner, 2012; Weidemann et al., 2012). Lethal congestive heart failure and supraventrivular arrhythmias is the primary mode of death in ~60% of patients with FRDA (Harding, 1981; Tsou et al., 2011; Weidemann et al., 2012).

The mutated gene in FRDA is localized on the long arm of chromosome 9 (9q21.11) and codes for a small mitochondrial protein called frataxin (FXN) (Campuzano et al., 1996, 1997; Koutnikova et al., 1997). All FRDA patients carry at least one allele with an expansion of a GAA-triplet repeat in the first intron of the FXN gene. Most patients are homozygous for this mutation, but a few patients (4%) are compound heterozygous for the GAA expansion and a classical mutation (nonsense, missense, deletions, insertions) leading to loss of FXN function (Campuzano et al., 1996; Cossee et al., 1999; Gellera et al., 2007). Normal chromosomes contain up to 40 GAA repeats, whereas disease-associated alleles contain 100 to more than 1500 GAA repeats, most commonly ~600–900. This GAA expansion leads to transcriptional silencing of FXN through a mechanism involving modifications of the chromatin structure of the locus, resulting in expression of a structurally and functionally normal frataxin but at levels that are estimated at ~5–30% of normal (reviewed in Gottesfeld, 2007; Schmucker and Puccio, 2010). As demonstrated in knockout animals, complete absence of frataxin leads to early embryonic death (Cossee et al., 2000). The rare non-GAA mutations in FXN that have been associated with FRDA lead to production of non-functional or partially functional proteins (Correia et al., 2008). In most cases, compound heterozygous patients are clinically indistinguishable from patients that are homozygous for the GAA expansions, but a few missense mutations (e.g., G130V, D122Y, R165P, L106S) in compound heterozygous patients cause atypical or milder clinical presentations (Cossee et al., 1999; Gellera et al., 2007).

The genetic basis of FRDA in humans raises challenges for modeling the disease in other species. Despite the difficulty in generating perfect FRDA models, a multitude of complementary models have been generated enabling significant advances in understanding the function of frataxin, the pathophysiology of the disease and some of the mechanisms implicated in GAA-based silencing (reviewed in Martelli et al., 2012b; Perdomini et al., 2013). Due to its high evolutionary conservation, the effect of FXN depletion has been modeled in diverse organisms, including yeast (Babcock et al., 1997; Foury and Cazzalini, 1997), invertebrates such as *C. elegans* (Vazquez-Manrique et al., 2006; Ventura et al., 2006; Zarse et al., 2007) and Drosophila (Anderson et al., 2005; Llorens et al., 2007), and in mice (Puccio et al., 2001; Miranda et al., 2002; Simon et al., 2004; Al-Mahdawi et al., 2006; Martelli et al., 2012a). However, due to the complexity of the clinical phenotype of individuals with FRDA and the species specificity in regulation of certain fundamental pathways, in particular iron metabolism, mouse models or mammalian cell culture models are probably better suited to understand the pathophysiological mechanisms involved in the disease.

## Iron dysregulation in friedreich ataxia

Early characterization of the pathophysiology in individuals with FRDA provided evidence of a link between frataxin deficiency and cellular iron metabolism dysregulation. Indeed, Lamarche and colleagues were the first to report the presence of granular iron deposits in cardiomyocytes of FRDA patients (Lamarche et al., 1980). After the discovery of the disease-causing gene, the generation of the yeast strain deficient for the yeast frataxin homolog, Yfh1, (ΔYfh1) showed that iron could accumulate in large amount within mitochondria (Babcock et al., 1997; Foury and Cazzalini, 1997). In mammals, mitochondrial iron accumulation and deposits were observed in the conditional mouse model reproducing the cardiac phenotype (MCK mouse) (Puccio et al., 2001). Iron metabolism dysregulation was also observed in heart autopsies of individuals with FRDA (Michael et al., 2006; Ramirez et al., 2012). Biochemical studies of heart biopsies also demonstrated a deficit in mitochondrial iron-sulfur (Fe-S) cluster-containing enzymes (aconitase and respiratory chain complexes I-III) (Rotig et al., 1997). Finally, the presence of markers of oxidative damage in blood and urine samples was reported (Emond et al., 2000; Schulz et al., 2000; Bradley et al., 2004), although contradictory results from patient data have been reported (Di Prospero et al., 2007; Myers et al., 2008; Schulz et al., 2009). Altogether, these observations led to the early assumption of a pathophysiological implication of iron-dependent pathways in FRDA.

The presence of mitochondrial iron accumulation in FRDA-affected neurons is however less clear. Both dentate nucleus and dorsal root ganglions (DRGs) of individuals with FRDA have been studied to investigate iron dysregulation. Dentate nucleus is an iron-rich cerebellar structure that shows signs of neurodegeneration in patients with FRDA (Koeppen, 2011). Despite a report of difference in the MRI signals that suggests an overall increase of iron in the dentate nucleus of individuals with FRDA (Boddaert et al., 2007), no difference in iron concentrations was measured using autopsies (Koeppen et al., 2007). However, modification of the expression of iron-related proteins such as transferrin receptor 1 (TFR1), ferritins (FRTs) and ferroportin (FPN) were observed, thereby suggesting a change in iron metabolism (Koeppen et al., 2007). Further investigations using X-ray fluorescence (XRF) suggested that iron was relocating from dying neurons to microglia of dentate nucleus (Koeppen et al., 2012). Similarly, DRGs from individuals with FRDA do not show overall iron concentrations above normal (Koeppen et al., 2009). However, the expression of FRTs, the iron-storage proteins, increases as satellite cells surrounding affected DRG neurons proliferate (Koeppen et al., 2009, 2013), thus suggesting again a redistribution of iron from dying neurons to satellite cells.

Although these observations suggest that iron is released during neuronal degeneration and then stored by surrounding glial cells, they do not give any indication on the primary involvement of iron dysregulation in the neuropathophysiology. In particular, mitochondrial iron deposits have never been reported in neurons from FRDA individuals and were not observed in an inducible conditional mouse model reproducing the neuronal phenotype (Prp mice) (Simon et al., 2004).

To understand the role and pathophysiological implication of iron in the disease, it is therefore essential to understand the function of frataxin and how its impairment can lead to cellular iron dysregulation.

## Iron and frataxin function

### Frataxin as an iron-binding protein

**Figure 1:**
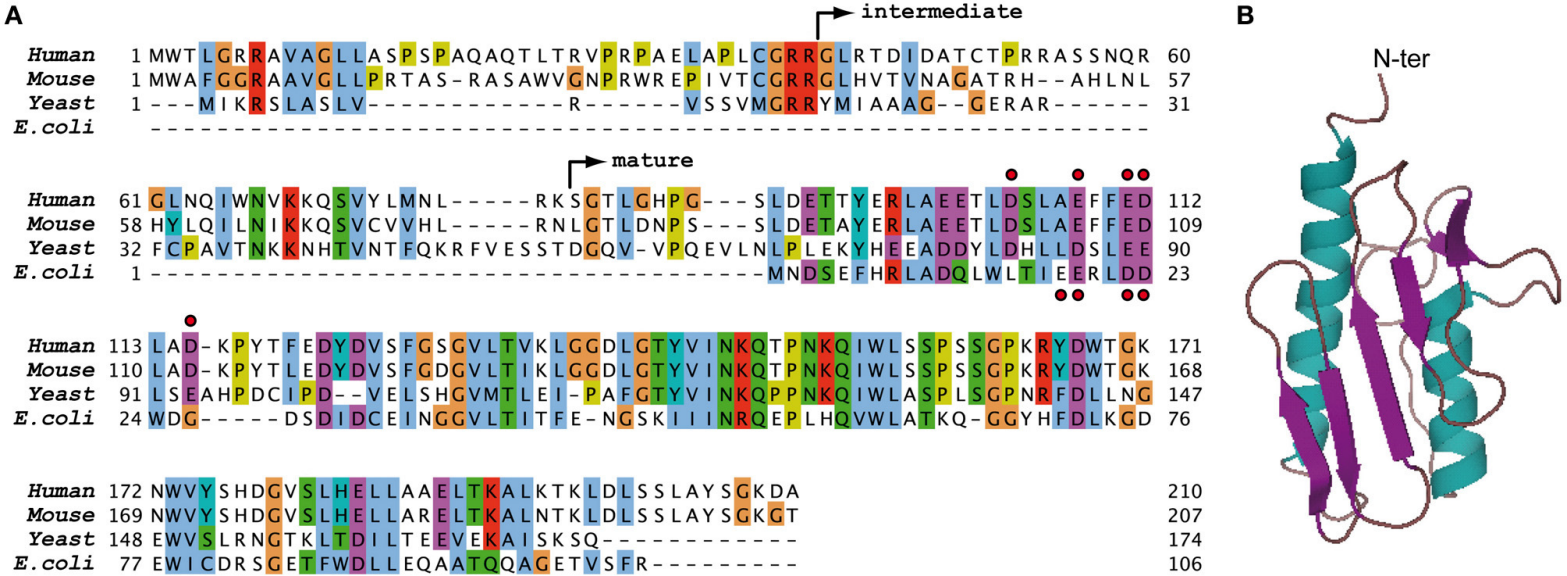
**Sequences and structure of frataxin. (A)** Sequence alignment of human, mouse, yeast, and bacterial frataxin. The non-conserved N-terminal parts of human, mouse, and yeast frataxins contain the mitochondrial targeting sequence of the protein. The arrows indicate the intermediate and mature forms of human FXN that are obtained during the two-step maturation process *in vivo* (Schmucker et al., 2008). The red dots indicate acidic residues of the N-terminal alpha helix of human and bacterial frataxins that are involved in the primary iron binding site of the monomeric protein. **(B)** Three dimensional structure of human frataxin obtained by NMR (1LY7) (Musco et al., 2000).

Frataxin is a highly conserved protein present from gram-negative bacteria to eukaryotes, including yeast and mammals (Figure 1A). Frataxin is localized within the eukaryotic mitochondria and is ubiquitously expressed in mammals. The structure of frataxin is unique and conserved in between species: frataxin is a small globular acidic protein composed of a long N-terminal alpha helix and a short C-terminal alpha helix that both interact with a central beta-sheet structure (Figure 1B) (Musco et al., 2000).

Although bacterial (CyaY), yeast (Yfh1), and mammalian (FXN) frataxins all exist as soluble monomers, early *in vitro* studies of bacterial CyaY and yeast Yfh1 showed that the proteins are able to form oligomeric spheroidal structures in the presence of excess iron (Adamec et al., 2000; Gakh et al., 2002; Layer et al., 2006; Adinolfi et al., 2009). These oligomeric structures can capture up to 50–75 atoms of iron, in a similar way as ferritin. Due to its property in scavenging iron, oligomeric frataxin was initially proposed to act as ferritins by providing bio-available iron within mitochondria (Adamec et al., 2000; Cavadini et al., 2002). This hypothesis was further sustained by the capacity of mitochondrial ferritin to complement for frataxin deficiency in the ΔYfh1 yeast strain and in HeLa cells (Campanella et al., 2004; Zanella et al., 2008). However, further experiments in yeast showed that expression of human mitochondrial ferritin only partially rescues the ΔYfh1 strain through a mechanism that does not overlap with frataxin function (Sutak et al., 2012). In addition, modulating the expression of Yfh1 in a yeast mutant strain that accumulates iron in mitochondria, in a similar way as the ΔYfh1 strains, does not modify iron bio-availability (Seguin et al., 2010). The relevance of the *in vivo* function of oligomeric frataxin is also questioned by *in vitro* data showing that bacterial CyaY forms iron-rich oligomeric structures only under aerobic conditions and high ionic strengths (Adinolfi et al., 2002; Layer et al., 2006). Furthermore, yeast Yfh1 bearing a point mutation that prevents oligomerization can rescue the ΔYfh1 strain (Aloria et al., 2004), therefore indicating that oligomerization is not required to fulfill the main function of frataxin *in vivo*.

In higher eukaryotes, the oligomerization process does not appear to be fully conserved. Frataxin is encoded by a nuclear gene and synthesized as a precursor protein (FXN^1−210^) that is then matured in two steps within the mitochondrial matrix to give an intermediate form (FXN^42−210^) and the major mature form (FXN^81−210^) (Condo et al., 2007; Schmucker et al., 2008) (Figure 1A). Only the precursor and intermediate forms of FXN can form oligomers in an iron-independent way, whereas mature human FXN is not prone to oligomerization (O'neill et al., 2005; Prischi et al., 2009). Furthermore, *in vivo* experiments using mouse fibroblasts deleted for the endogenous murine frataxin showed that the expression of the mature human FXN^81−210^ is sufficient to promote cell survival (Schmucker et al., 2011), thus indicating that oligomerization is not a process required for the primary and essential function of mammalian frataxin *in vivo*.

Although the functional relevance of an iron-rich oligomeric frataxin is questionable, there is clear evidence that monomeric frataxin can also bind iron *in vitro*. Several iron-binding sites have been characterized depending on the oxidative state of iron (Fe^2+^ or Fe^3+^) and the origin of the frataxin proteins (CyaY, Yfh1, or human FXN) (Yoon and Cowan, 2003; Bou-Abdallah et al., 2004; Cook et al., 2006; Yoon et al., 2007; Huang et al., 2008). A primary iron-binding site appears however to be conserved and involves residues of the acidic ridge localized within the first alpha helix of frataxin (Figure 1). The site binds Fe^2+^ with a dissociation constant (Kd) within the micromolar range (3–55 μ M) (Yoon and Cowan, 2003; Nair et al., 2004; Cook et al., 2006) but seems to be poorly specific as other cations were shown to also bind CyaY (Pastore et al., 2007).

### Function of frataxin in Fe-S cluster biogenesis

The capacity of frataxin to bind iron and the evidence of an iron metabolism dysregulation in individuals with FRDA and in ΔYfh1 yeast strains led to the assumption that frataxin plays a key role in the mitochondrial iron metabolism. Further biochemical and interaction studies provided several hypotheses. Interactions with mitochondrial aconitase, ferrochelatase and proteins of the mitochondrial Fe-S cluster machinery were reported (Gerber et al., 2003; Bulteau et al., 2004; Yoon and Cowan, 2004; Bencze et al., 2007), and the hypothesis of frataxin being an iron provider to various iron-dependent mitochondrial pathways was brought forward. However, interactions with aconitase and ferrochelatase are still poorly characterized and were reported not to be reproducible (Schmucker et al., 2011). To date, only the interaction of frataxin with proteins involved in the mitochondrial Fe-S biogenesis have been extensively and convincingly characterized.

**Figure 2:**
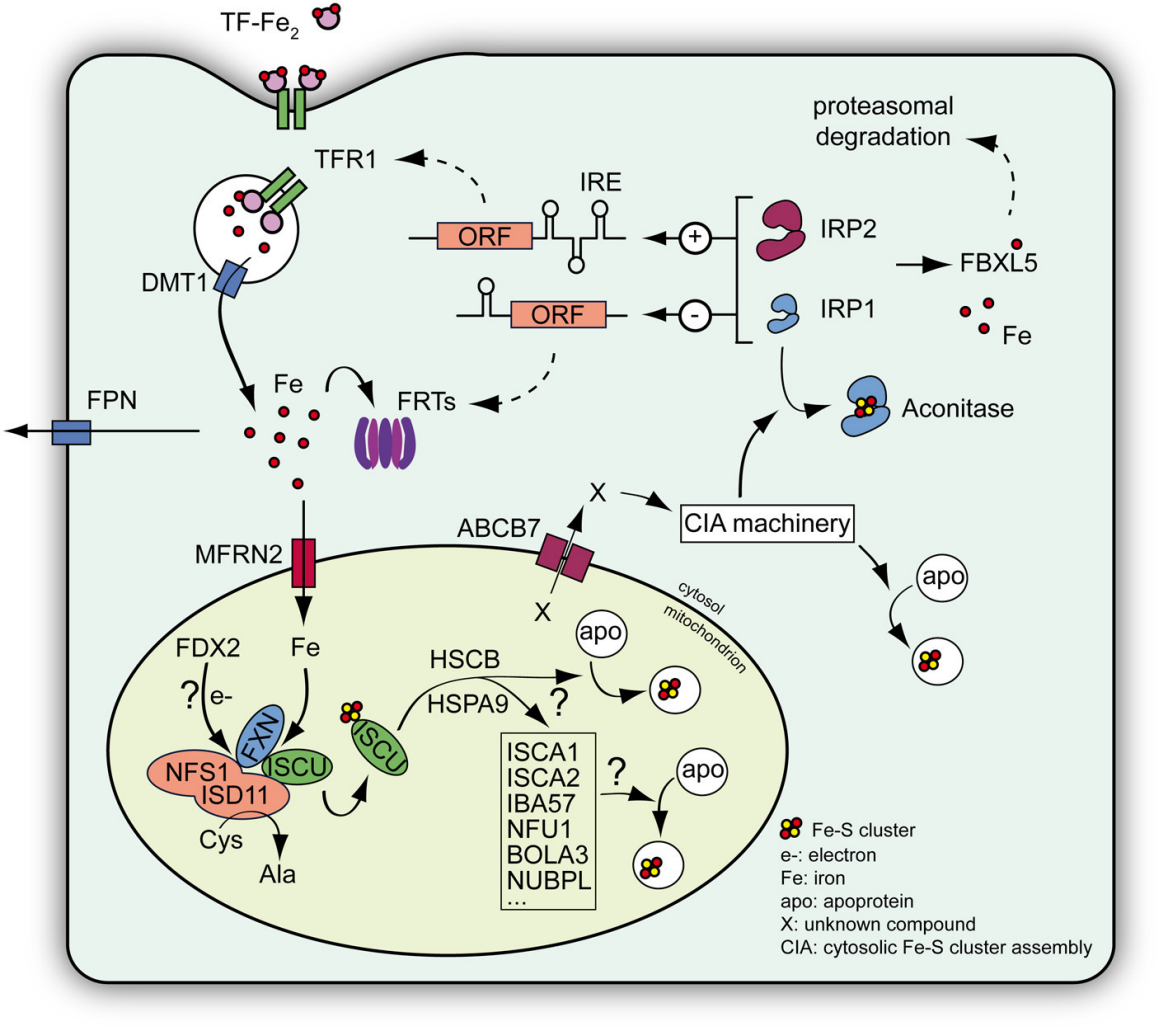
**Schematic view of the Fe-S cluster machinery and the IRP-mediated cellular iron regulation**. *De novo* Fe-S cluster biogenesis occurs within mitochondria and involves assembly of inorganic sulfur and iron on a scaffold protein ISCU. Iron is imported into the mitochondria by mitoferrins (MFRN). The process of Fe-S cluster assembly occurs within a complex consisting of NFS1-ISD11, the cysteine desulfurase providing the sulfur, ISCU and eventually frataxin (FXN), which regulates the NFS1 activity and the entry of iron within the complex (Colin et al., 2013). The process also needs electrons (e–) that may be provided by a mitochondrial ferredoxin (FDX2). Once assembled the cluster on ISCU is transferred to acceptor proteins with the help of additional proteins, such as the chaperones HSCB and HSPA9, ISCA proteins, IBA57, NFU1, BOLA3, and NUBPL (reviewed in Lill, 2009; Beilschmidt and Puccio, 2014). Alternatively, a still uncharacterized compound (X) provided by the mitochondrial machinery is exported to the cytosol via ABCB7 and is used by the cytosolic Fe-S cluster assembly machinery (CIA machinery) to assemble Fe-S clusters for cytosolic and nuclear acceptor proteins. Among the cytosolic Fe-S cluster acceptors, IRP1 is a regulator of cellular iron metabolism. In normal conditions, IRP1 binds a Fe-S cluster to become an aconitase devoid of regulatory capacity. IRP2 exists only as an apoprotein and is regulated through proteasomal degradation mediated by the iron sensor protein FBXL5 (Salahudeen et al., 2009; Vashisht et al., 2009). Both IRPs can regulate the expression of key genes involved in iron metabolism, such as transferrin receptor 1 (TFR1), ferritins (FRTs), and the iron exporter ferroportin (FPN), by binding a specific mRNA motif called IRE. Depending on the location of the IRE compared to the open reading frame (ORF), IRPs can increase (+) or decrease (−) protein expression, thus controlling cellular iron import and storage (reviewed in Anderson et al., 2012). DMT1: divalent metal transporter involved in iron import.

Fe-S clusters are inorganic redox-active protein cofactors that are present in almost all living organisms. They play cardinal roles in various functions throughout the cell, including electron transport in the respiratory complexes and DNA repair or metabolism. Although Fe-S clusters can adopt different configurations, [Fe_2_S_2_] and [Fe_4_S_4_] clusters are the most frequent Fe-S clusters in eukaryotes. *De novo* biosynthesis of Fe-S clusters occurs within mitochondria (reviewed in Lill, 2009; Beilschmidt and Puccio, 2014). The first step involves the assembly of a Fe-S cluster on a scaffold protein ISCU (Isu in yeast) from inorganic iron and sulfur. A cysteine desulfurase complex NFS1/ISD11 provides the sulfur through a persulfide intermediate. ISCU and NFS1/ISD11 interact and form a ternary ISCU/NFS1/ISD11 complex with a most likely α2β 2γ4 stoichiometry (Schmucker et al., 2011; Colin et al., 2013). Once the cluster is assembled on ISCU, it is transferred to acceptor proteins with the help of additional components of the mitochondrial Fe-S cluster machinery, such as the HSCB/HSPA9 chaperone system or proteins (e.g., ISCA1/2) that provide Fe-S cluster to a subset of mitochondrial proteins (Figure 2). Alternatively, a still uncharacterized intermediate provided by the early Fe-S cluster machinery is exported from the mitochondria to the cytosol via the ABCB7 transporter where it is used by the cytosolic Fe-S cluster assembly machinery (CIA machinery) to generate Fe-S clusters for cytosolic and nuclear acceptor proteins (reviewed in Lill, 2009) (Figure 2).

**Figure 3:**
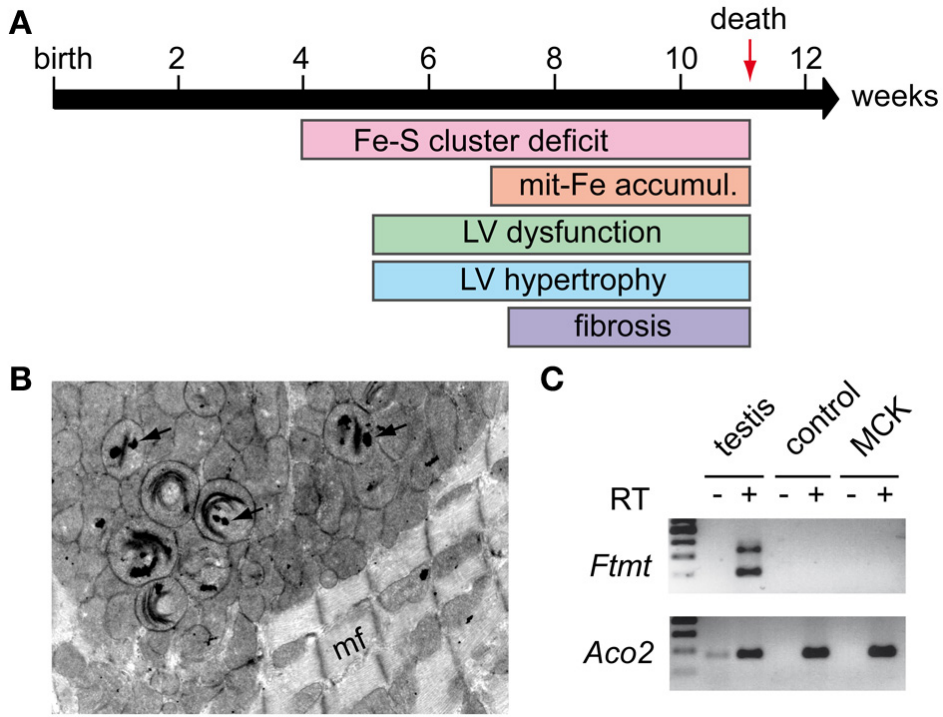
**MCK mouse model. (A)** Phenotypic characteristics of the MCK mouse model: MCK mice develop progressive hypertrophic cardiomyopathy characterized by progressive left ventricule (LV) dysfunction and hypertrophy starting around 5 weeks of age. The cardiomyopathy leads to cell death and fibrosis. MCK mice prematurely dye around 11 weeks. Fe-S cluster deficit is a primary feature in the mouse pathology with significant differences observed in 4 weeks old mice. Mitochondrial iron (mit-Fe) accumulation is observed in the later stage of the disease. **(B)** Electron microscopy picture obtained from a heart sample of a 7 weeks old MCK mouse showing mitochondrial abnormalities, in particular collapse cristae and electron-dense deposits (arrows) corresponding to mitochondrial iron deposits. mf, myofiber. **(C)** Semi-nested PCR on cDNA from heart samples of 8 weeks old control and deleted MCK mice was performed as described (Santambrogio et al., 2007) to assess mitochondrial ferritin (FTMT) expression. Total RNA from tissue was extracted using Trizol® reagent (Life Technologies) and submitted (+) or not (−) to reverse transcription (RT). Testis cDNA was used as positive control for *Ftmt* expression, and a classical PCR to amplify mitochondrial aconitase (Aco2) cDNA was carried out as a control for reverse transcription and loading. Samples without reverse transcription (RT–) were used as control for specific PCR amplification.

The first hints for the primary involvement of frataxin in Fe-S cluster biogenesis came from the characterization of the cardiac mouse model mimicking the FRDA cardiomyopathy, as Fe-S cluster dependent enzymes were affected prior to the appearance of the heart dysfunction and the mitochondrial iron accumulation (Puccio et al., 2001; Seznec et al., 2004; Martelli et al., 2007) (Figure 3A). In parallel, early phylogenetic studies predicted a role of frataxin in Fe-S cluster metabolism (Huynen et al., 2001). The implication of frataxin in Fe-S cluster biogenesis was later confirmed in yeast depleted for Yfh1 (Duby et al., 2002; Muhlenhoff et al., 2002). Furthermore, an iron-dependent interaction of Yfh1 with Nfs1 and Isu1 was reported in yeast (Gerber et al., 2003), while *in vitro* reconstitution experiments showed that human FXN could transfer iron to ISCU (Yoon and Cowan, 2003) and that bacterial CyaY could provide iron for Fe-S cluster formation (Layer et al., 2006). Altogether, these results suggested that frataxin might be the iron donor for the assembly of the Fe-S cluster *in vivo*. However, data from mammals, yeast, and bacteria were quite controversial as to the direct frataxin protein partner in Fe-S biogenesis (Gerber et al., 2003; Layer et al., 2006; Shan et al., 2007; Li et al., 2009). Recently, these results were reconciled by independent work using mammalian recombinant proteins showing that frataxin interacts with a preformed complex composed of NFS1, ISCU, and ISD11 (Tsai and Barondeau, 2010; Schmucker et al., 2011). A similar complex was also reported in bacteria (Prischi et al., 2010). In line with results obtained with the bacterial CyaY suggesting that frataxin is a regulator of the Fe-S clusters synthesis (Adinolfi et al., 2009), the binding of frataxin in the mammalian system was shown to stabilize the complex and to activate the cysteine desulfurase activity (Tsai and Barondeau, 2010; Colin et al., 2013). Moreover, although the formation of the complex was shown to be iron-independent (Schmucker et al., 2011), frataxin appears to concomitantly activate the cysteine desulfurase activity and to control iron entry within the complex (Colin et al., 2013). More recently, the biochemical characterization of the successive steps of the cysteine desulfurase activity in yeast provided evidence that frataxin triggers a conformational change that modifies the substrate-binding site of the enzyme (Pandey et al., 2013). All these results therefore indicate that frataxin, by controlling both iron entry and sulfide production, is essential in the process of Fe-S cluster assembly during the initial stage of the biogenesis. However, how frataxin controls iron entry within the complex still need to be determined. In particular, residues that define the primary iron-binding site of the protein *in vitro* were also shown to be involved in the interaction with the cysteine desulfurase and/or the ISCU/NFS1/ISD11 complex (Prischi et al., 2010; Schmucker et al., 2011). Hence, the *in vivo* implication of this iron-binding site needs to be further investigated.

## From iron-sulfur cluster deficit to mitochondrial iron accumulation

Although the recent advances point to a primary role of frataxin in Fe-S cluster biogenesis, the cellular mechanism that links frataxin deficiency to mitochondrial iron overload remains elusive. However, mitochondrial iron accumulation is not specific to frataxin deficiency, but rather appears as a general hallmark of primary Fe-S deficiency, as it has been observed in various yeast strains deleted for different genes involved in Fe-S cluster biogenesis (Kispal et al., 1997; Garland et al., 1999; Schilke et al., 1999; Lange et al., 2000; Voisine et al., 2001). Furthermore, mutations in human genes implicated in Fe-S cluster biogenesis have recently been identified as disease-causing genes (reviewed in Beilschmidt and Puccio, 2014), and some of the associated disorders are also characterized by mitochondrial iron accumulation. Mutations in the scaffold protein ISCU lead to myopathy with lactic acidosis with different severity, also known as Swedish myopathy (Mochel et al., 2008; Olsson et al., 2008; Kollberg et al., 2009). The major mutation, due to a founder effect in Sweden, induces a muscle-specific cryptic splice site that leads to a truncated protein (Mochel et al., 2008; Olsson et al., 2008). In muscle biopsies, iron labeling (Perl's staining) showed accumulation of iron within mitochondria (Mochel et al., 2008; Kollberg et al., 2009). Mutations in ABCB7 are associated with X-linked sideroblastic anemia with ataxia, a condition that is characterized by the presence of iron-rich perinuclear mitochondria within erythroblasts (sideroblasts) (Allikmets et al., 1999; Bekri et al., 2000). Similarly, a mutation in GLRX5, a protein linked to Fe-S cluster biogenesis, although its function is still unclear (Rodriguez-Manzaneque et al., 2002), was identified in a patient presenting sideroblastic anemia (Camaschella et al., 2007). Interestingly, mutations linked to human disease in proteins involved in the delivery of Fe-S cluster to a subset of mitochondrial proteins (e.g., NFU1, IBA57, BOLA3—see Figure 2) are not associated with iron accumulation (reviewed in Beilschmidt and Puccio, 2014).

It is most likely that the pathways leading to iron dysregulation and mitochondrial iron accumulation are shared among the different disease linked to primary Fe-S cluster biogenesis. Although the link between Fe-S cluster deficit and iron metabolism has been observed in both yeast and mammals, it is unlikely that the mechanisms involved in mitochondrial iron accumulation are strictly conserved between the two species, as cellular iron homeostasis involves different modes of regulation in yeast and higher eukaryotes.

### Cellular iron regulation in mammals

In mammals, the Iron Regulatory Proteins (IRP) 1 and 2 largely regulates cellular iron homeostasis. IRP1 and IRP2 are cytosolic translational regulators that control the expression of proteins involved in iron handling and distribution (Figure 2), as well as targeting other transcripts that are not directly involved in iron metabolism such as HIF2α and mitochondrial aconitase (reviewed in Hentze et al., 2010; Anderson et al., 2012). IRP1 and 2 can bind specific mRNA motifs, called Iron Responsive Elements (IRE), thereby influencing protein expression by regulating either protein translation or mRNA metabolism. Indeed, when the IRE is located in the 5′ UTR, binding of IRPs blocks translation, whereas the formation of an IRP/IRE complex in the 3′ UTR leads to an increase half-life of the mRNA, therefore increasing translation. Transferrin receptor 1 (TFR1), implicated in cellular iron import, and the ferritins (FRTs), involved in cytosolic iron storage, are key proteins regulated by IRPs (Figure 2). The mRNA of TFR1 contains several IRE motifs within the 3′ UTR, whereas mRNAs coding for FRTs contain an IRE motif in the 5′ UTR. Although IRP1 and IRP2 exhibit some functional redundancy as both proteins can control TFR1 and FRTs expressions, some IRE sequence specificities have been reported (Ke et al., 1998; Anderson et al., 2013). However, the activity of IRP1 and IRP2 are mostly regulated differently. When cytosolic iron concentration increases, the iron-binding protein FBXL5 targets IRP2 to ubiquitination and proteasomal degradation (Figure 2) (Salahudeen et al., 2009; Vashisht et al., 2009). However, although IRP1 can also be targeted by FBXL5, its IRE-binding activity is mainly negatively regulated through the insertion of a [Fe_4_-S_4_] cluster leading to a protein with cytosolic aconitase activity devoid of IRE-binding activity (Figure 2) (Haile et al., 1992a,b).

IRP1 and IRP2 have been shown to have overlapping functions as observed in knockout mouse models (Galy et al., 2005; Anderson et al., 2013; Ghosh et al., 2013), however, IRP2 is considered as the main iron regulator under normal physiological conditions as IRP1 exists mainly as an aconitase (Meyron-Holtz et al., 2004; Moroishi et al., 2011) (Figure 2).

### The nature of the mitochondrial accumulated iron

To date, no data provide a clear answer on the nature of the iron that is accumulated in affected mitochondria, but few interesting hints are available. The analysis of heart tissues from MCK mice by electron microscopy showed the presence of electron-dense particles within the mitochondrial matrix (Figure 3B) that correlated with iron accumulation (Puccio et al., 2001). Similar structures were observed in mitochondria of heart tissue from individuals with FRDA (Michael et al., 2006) and in the liver conditional knockout mouse (ALB mouse) (Martelli et al., 2012a). In FRDA patients' samples, histological analysis suggested that mitochondrial ferritin (FTMT) might be involved in the formation of the iron-rich structures (Michael et al., 2006). However, recent data obtained in MCK mice suggested that FTMT is not involved since iron was reported to be mostly present as mineral non-ferritin aggregates (Whitnall et al., 2012). In addition, despite a similar pattern of iron deposits as in patients (Figure 3B), no Ftmt mRNA could be detected in both the heart of MCK mice (Figure 3C) or the liver of ALB mice (AM and HP, unpublished results) using a semi-nested PCR protocol developed to specifically assess Ftmt expression (Santambrogio et al., 2007). These results further question the potential role of FTMT in the molecular pathophysiology.

Interestingly, the iron-rich aggregates observed in mouse models and patient samples are reminiscent of the mitochondrial phosphate-iron nano-particles that were identified in ΔYfh1 yeasts (Lesuisse et al., 2003), as well as in the yeast strains deleted for Yah1 and Atm1 (the homologs of ferredoxin or ABCB7, respectively) (Miao et al., 2008, 2009). The formation of these aggregates in yeast mitochondria lead to a decrease of available iron that affects heme biosynthesis (Lesuisse et al., 2003; Seguin et al., 2010).

### Modifications of iron-related protein and gene expression in the cardiac mouse model

Although the characterization of the cardiac MCK mouse model and cellular models deficient in frataxin have provided several clues on the nature of iron dysregulation occurring after frataxin deficiency (Seznec et al., 2005; Whitnall et al., 2008, 2012; Huang et al., 2009), available data are sometimes contradictory.

The variations in activity, protein and mRNA expressions of key genes implicated in iron regulation and distribution that have been reported in MCK mice are shown in Table 1. IRP1 was shown to be activated into its IRE-binding form in MCK mice (Seznec et al., 2005), in agreement with the primary role of frataxin in Fe-S cluster biogenesis. This was also observed in frataxin knockdown experiments using HeLa cells (Stehling et al., 2004). Accordingly, similar observations were made in knockdown experiments or knockout animals targeting other proteins of the Fe-S cluster machinery (Biederbick et al., 2006; Fosset et al., 2006; Pondarre et al., 2006; Wang et al., 2007). Furthermore, ferritin L (FRTL) mRNA and protein levels were shown to progressively increase in MCK mice (Seznec et al., 2005). More recently, an increase of transferrin receptor 1 (TFR1) protein expression concomitant to a decrease of expression of ferroportin (FPN), the cellular iron exporter, was reported in FXN-deficient mice (Whitnall et al., 2008; Huang et al., 2009), thus suggesting an overall increase of the iron import capacity. This modification of iron import was confirmed by ^59^Fe import experiments (Whitnall et al., 2008). However, in contradiction with the reported increase of FRTL levels (Seznec et al., 2005), both ferritin L and H displayed decreased protein levels when compared to control animals, and most strikingly, no difference in IRP1 IRE-binding activity could be observed (Whitnall et al., 2008, 2012). As iron metabolism is a tightly regulated pathway, caution in comparing animal data raised in different laboratories have to be taken [differential animal food, circadian rhythm, and experimental condition before sacrifice (diet intake)]. Notably, in the latter reports, the mobility shift assays show that IRP1 is almost fully activated into its IRE-binding form in both control and deleted mice (Whitnall et al., 2008, 2012), in contradiction with IRP1 being essentially an aconitase in normal physiological conditions. Furthermore, the authors also provided evidence that IRP2 is more active in FXN-deleted animals (Whitnall et al., 2008, 2012), thus indicating a depletion of iron within the cytosol leading to reduced proteasomal degradation of IRP2. Cytosolic iron depletion was confirmed by iron concentration measurements after tissue fractionation (Whitnall et al., 2008), although these measurements do not discriminate between the available cytosolic iron pool and the one trapped within ferritins, which were shown under the same experimental conditions to be decreased (Whitnall et al., 2008). More interestingly, the mRNA level of mitoferrin-2 (MFRN2), the mitochondrial iron transporter, is significantly increased in MCK mice (Huang et al., 2009). A similar increase of MFRN2 mRNA has been observed in skeletal muscle biopsies from ISCU myopathy patients (Crooks et al., 2014). These results suggest the existence of a Fe-S cluster-dependent regulation of mitochondrial iron import, whether direct or indirect, that may control mitochondrial iron overload. However, whether up-regulation of MFRN2 is sufficient to explain mitochondrial iron accumulation in FXN-deficient mice is not known.

**Table 1:**
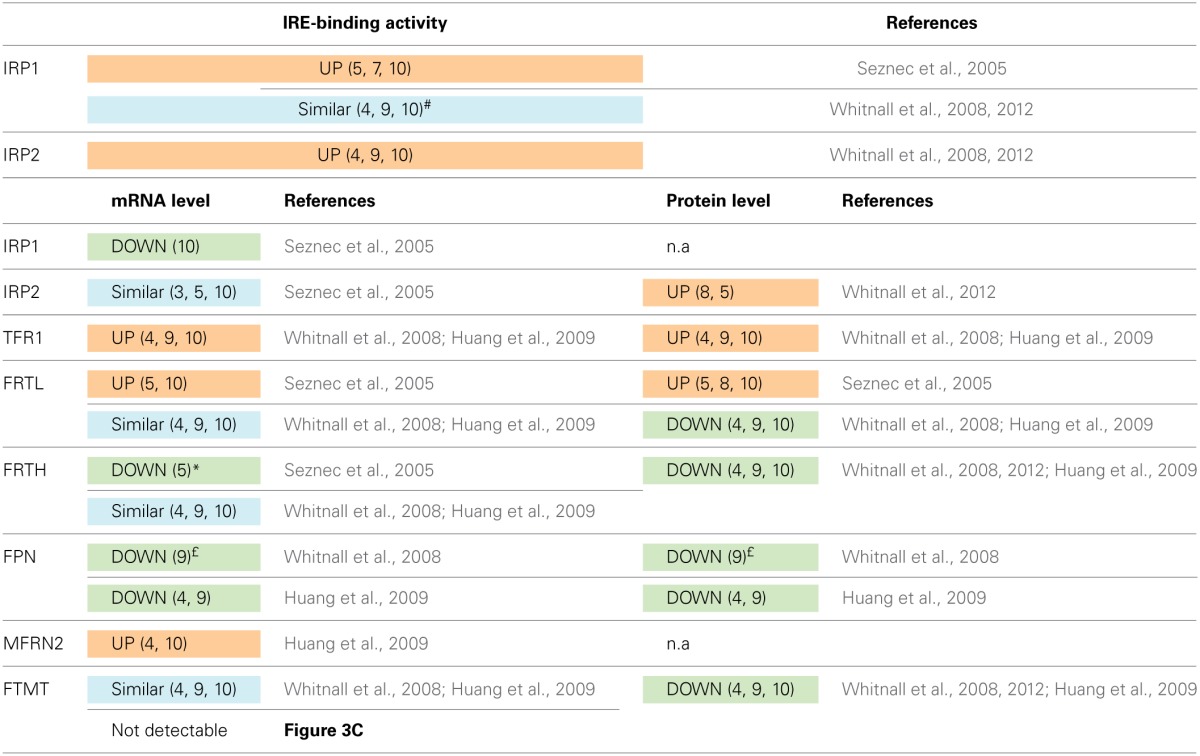
**Modifications in iron-related gene expression and activity in MCK mice**.

*“UP” indicates increase of expression or activity in Fxn-deleted mice compared to control mice, “DOWN” means decrease and “Similar,” no detectable change. Ages (in weeks) corresponding to the observations are indicated in brackets*.

*^#^IRP1 almost fully activated in both control and deleted MCK mice*.

^*^Mitochip array data; n.a: data not available.

*^£^No difference was observed at 4 weeks*.

## Conclusion and perspectives

To understand the consequence of mitochondrial iron overload on the pathophysiology of FRDA is of particular interest in the context of therapeutic approaches for FRDA. Early report suggested that the iron accumulation generated toxic free radicals through Fenton reaction, therefore implying iron chelators as possible therapeutic agents. Although it is most likely that reactive oxygen species play a role in FRDA, the primary involvement as well as the importance of reactive oxygen species in the pathophysiology are still a matter of debate in the field.

Recently, Deferiprone, an iron chelator that may cross the blood brain barrier, has been used in preclinical and clinical studies for FRDA, but the results were somehow puzzling as different doses showed opposite effects, if any (reviewed in Pandolfo and Hausmann, 2013), therefore further questioning the rationale behind the use of chelation therapy in FRDA. In line with these results, the data obtained with the MCK mouse model further indicates that both cellular and mitochondrial iron imports are increased in the absence of frataxin. Does it mean that cells, and in particular mitochondria, are in iron deprivation rather than facing toxic iron accumulation? This question may seem counterintuitive when total mitochondrial iron is measured in FRDA models, but the characterization of the accumulated iron in frataxin-deficient cells provide further evidence that iron may not be biologically available within mitochondria. Hence, the role of chelation therapies should be to target this non-available iron to make it available again for biological processes, rather than depleting iron from the cell as it is expected in other disorders of iron overload.

The primary function of frataxin in Fe-S cluster biogenesis is now on the way of being fully elucidated through the biochemical characterization of the complex in which it is involved. Attention is also brought to the understanding of the cellular consequences of frataxin deficiency. In particular, the mechanism leading to mitochondrial iron accumulation, and most importantly, the consequences of this accumulation on the pathophysiology is under investigation. All together, these data will be valuable for the evaluation and design of new therapeutic approaches that may (or not) use iron chelators. The recent identification of several other human genetic disorders linked to primary Fe-S cluster deficiency and displaying mitochondrial iron accumulation, as well as the development of the corresponding cellular and animal models will clearly be an asset to address these questions.

### Conflict of interest statement

The authors declare that the research was conducted in the absence of any commercial or financial relationships that could be construed as a potential conflict of interest.
